# Detection and margin assessment of thyroid carcinoma with microscopic hyperspectral imaging using transformer networks

**DOI:** 10.1117/1.JBO.29.9.093505

**Published:** 2024-07-24

**Authors:** Minh Ha Tran, Ling Ma, Hasan Mubarak, Ofelia Gomez, James Yu, Michelle Bryarly, Baowei Fei

**Affiliations:** a University of Texas at Dallas, Department of Bioengineering, Richardson, Texas, United States; b University of Texas at Dallas, Center for Imaging and Surgical Innovation, Richardson, Texas, United States; c University of Texas Southwestern Medical Center, Department of Radiology, Dallas, Texas, United States

**Keywords:** hyperspectral imaging, transformer, thyroid carcinoma, margin detection, microscope

## Abstract

**Significance:**

Hyperspectral imaging (HSI) is an emerging imaging modality for oncological applications and can improve cancer detection with digital pathology.

**Aim:**

The study aims to highlight the increased accuracy and sensitivity of detecting the margin of thyroid carcinoma in hematoxylin and eosin (H&E)-stained histological slides using HSI and data augmentation methods.

**Approach:**

Using an automated microscopic imaging system, we captured 2599 hyperspectral images from 65 H&E-stained human thyroid slides. Images were then preprocessed into 153,906 image patches of dimension 250×250×84  pixels. We modified the TimeSformer network architecture, which used alternating spectral attention and spatial attention layers. We implemented several data augmentation methods for HSI based on the RandAugment algorithm. We compared the performances of TimeSformer on HSI against the performances of pretrained ConvNext and pretrained vision transformers (ViT) networks on red, green, and blue (RGB) images. Finally, we applied attention unrolling techniques on the trained TimeSformer network to identify the biological features to which the network paid attention.

**Results:**

In the testing dataset, TimeSformer achieved an accuracy of 90.87%, a weighted $F_{1}$ score of 89.79%, a sensitivity of 91.50%, and an area under the receiving operator characteristic curve (AU-ROC) score of 97.04%. Additionally, TimeSformer produced thyroid carcinoma tumor margins with an average Jaccard score of 0.76 mm. Without data augmentation, TimeSformer achieved an accuracy of 88.23%, a weighted $F_{1}$ score of 86.46%, a sensitivity of 85.53%, and an AU-ROC score of 94.94%. In comparison, the ViT network achieved an 89.98% accuracy, an 88.14% weighted $F_{1}$ score, an 84.77% sensitivity, and a 96.17% AU-ROC. Our visualization results showed that the network paid attention to biological features.

**Conclusions:**

The TimeSformer model trained with hyperspectral histological data consistently outperformed conventional RGB-based models, highlighting the superiority of HSI in this context. Our proposed augmentation methods improved the accuracy, the $F_{1}$ score, and the sensitivity score.

## 1 Introduction

Thyroid cancer is one of the fastest-growing types of cancer in the United States with an estimated 43,720 new cases registered in 2023 alone.^1^ Even though a vast majority (more than 90%) of thyroid nodules are benign, some patients would develop malignant tumors that can migrate to other organs.^2^ The most common types of thyroid cancer originate from follicular epithelial cells. This includes papillary thyroid cancer (80% to 85% of cases^3^), follicular thyroid cancer, and anaplastic thyroid cancer.^2^ Medullary thyroid cancer, which accounts for only 1% to 2% of all thyroid cancer cases, originates from the parafollicular C cells.^2^ Thyroid lobectomy is a common treatment option if immediate risk is detected. During surgery, a pathologist determines the border between benign and cancerous tissue on a frozen section and helps identify the positive margin under a microscope. If cancer remains present at the edge of the tissue, the margin is labeled as positive, suggesting that the tumor was not entirely resected. The acceptable margin for thyroid carcinoma ranges from 5 to 20 mm.^4^ However, the thyroid region is vital for the quality of life; thus, it is also important to preserve as many non-tumor cells as possible. A balance is needed between a good negative margin and a good living outcome, and correct determination of the margin helps.

Machine learning on red, green, and blue (RGB) histopathology images has been a hot topic for researchers for the last 10 years.^5^ The goal of automated detection is to generate decision margins comparable to that of trained pathologists at a fraction of the time and resources. Halicek et al.^6^ used a convolutional neural network (CNN) to classify malignant thyroid images against regular ones with an accuracy of 89.4% and an area under the receiving operator characteristic curve (AU-ROC) value of 0.954. Range et al.^7^ reported using CNN to achieve an AU-ROC value of 0.932. Dov et al.^8^ used weakly supervised learning to predict thyroid malignancy with an AU-ROC value of 0.870. Researchers are continuing to improve the performances of machine learning systems by either introducing more sophisticated learning methods or using novel imaging modalities. Hyperspectral imaging (HSI) is an optical imaging technique that captures more spectral information than RGB cameras. HSI has been used on hematoxylin and eosin (H&E)-stained slides to improve classification results.^9^^–^^16^ Our previous works^11^^,^^15^ showed that using hyperspectral histology images of head and neck cancer slides to train neural networks can result in improvements in accuracy compared with RGB images. Sun et al.^17^ developed deep neural networks to classify H&E-stained hyperspectral images of cholangiocarcinoma pathology. Ma et al.^13^ performed a pixel-level margin assessment on head and neck squamous cell carcinoma and achieved a median Hausdorff distance of 1.03 mm. Zhou et al.^18^ combined HSI and polarized light imaging for the visualization of head and neck carcinoma. However, many challenges face the development of neural networks for HSI, such as the lack of high-quality hyperspectral datasets and interpretable results. In addition, it is unclear why deep neural networks produce better classification results when trained on hyperspectral data over RGB and what wavelengths contribute to these improvements.

Typically, CNN is a common method for image classification. CNN uses convolutional filters to extract features in the spatial and spectral domains. In recent years, vision transformer (ViT) was used as an alternative to CNN to improve classification results on large datasets.^19^ Compared with CNN, ViT used the multi-head attention module to extract features. Each attention head aims to highlight different features using the attention mechanism. In remote sensing, researchers have explored using a transformer for the task of HSI classification.^20^^–^^24^ However, applications of transformer networks and HSI in the field of biomedical imaging are relatively new. Zeng et al.^24^ used a fusion of CNN and the attention layer to classify nephropathy H&E-stained slides. Li et al.^23^ converted the multi-head mechanism into a multi-band attention mechanism and classified cholangiocarcinoma histology samples.

Training transformer networks requires large amounts of data to overcome the lack of inductive bias. With HSI, it is not often feasible to produce data at such a scale. To overcome this, one can use pretrained networks (networks that have been trained on a vast number of datasets by other research or commercial institutions) or data augmentation (artificially inflating the existing dataset by modifying the training images).^25^ Steiner et al.^25^ found that, using proper augmentation techniques to train ViTs, one can achieve similar validation accuracy with only 50% of the original data. Several data augmentation methods for histology data have been developed. Tellez et al.^26^ and Faryna et al.^27^ proposed augmentation of hematoxylin-eosin-DAB contents. They achieved this using a known matrix of RGB responses of different types of stains to estimate the amount of stain within each image. However, when the number of spectra is large, their methods fail to estimate the spectral responses. Örnberg et al.^28^ proposed augmentation of spectral data with a popular augmentation technique called RandAugment;^29^ however, their transformations did not include spatial transformations and were not tested on a large image dataset.

In this paper, we propose using a pretrained video classifier network called TimeSformer, which is a state-of-the-art architecture for video classification.^30^ In TimeSformer, there are two alternating attention layers: a spatial attention layer and a temporal attention layer. Because both hyperspectral images and videos have an additional dimension that is orthogonal to the spatial dimension, we believe that a pretrained video classification network can be minimally modified to classify hyperspectral images. He et al.^31^ studied 3D deep CNN for HSI classification and showed skepticisms that pretrained CNN video classifiers can be used for HSI. To address this, we use a transformer network, which has less inductive bias (narrow assumptions about the data that influenced the trained network) compared with CNN.^19^ To our knowledge, we are the first to report the use of a transformer network with separate spectral and spatial attention layers for the classification of biological hyperspectral images. We propose new augmentation methods for RandAugment in the spectral domain, which are based on real physical phenomena and on non-negative factorization (NMF) decomposition.

The contributions of this study include the following: (1) we modify the temporal attention mechanism in pretrained TimeSformer and improve the accuracy and sensitivity of HSI classification, (2) we propose and test new data augmentation techniques specific for hyperspectral images, and (3) we report a large dataset of whole-slide hyperspectral images (WSHSIs).

## 2 Materials and Methods

### 2.1 Acquisition System

**Fig. 1:**
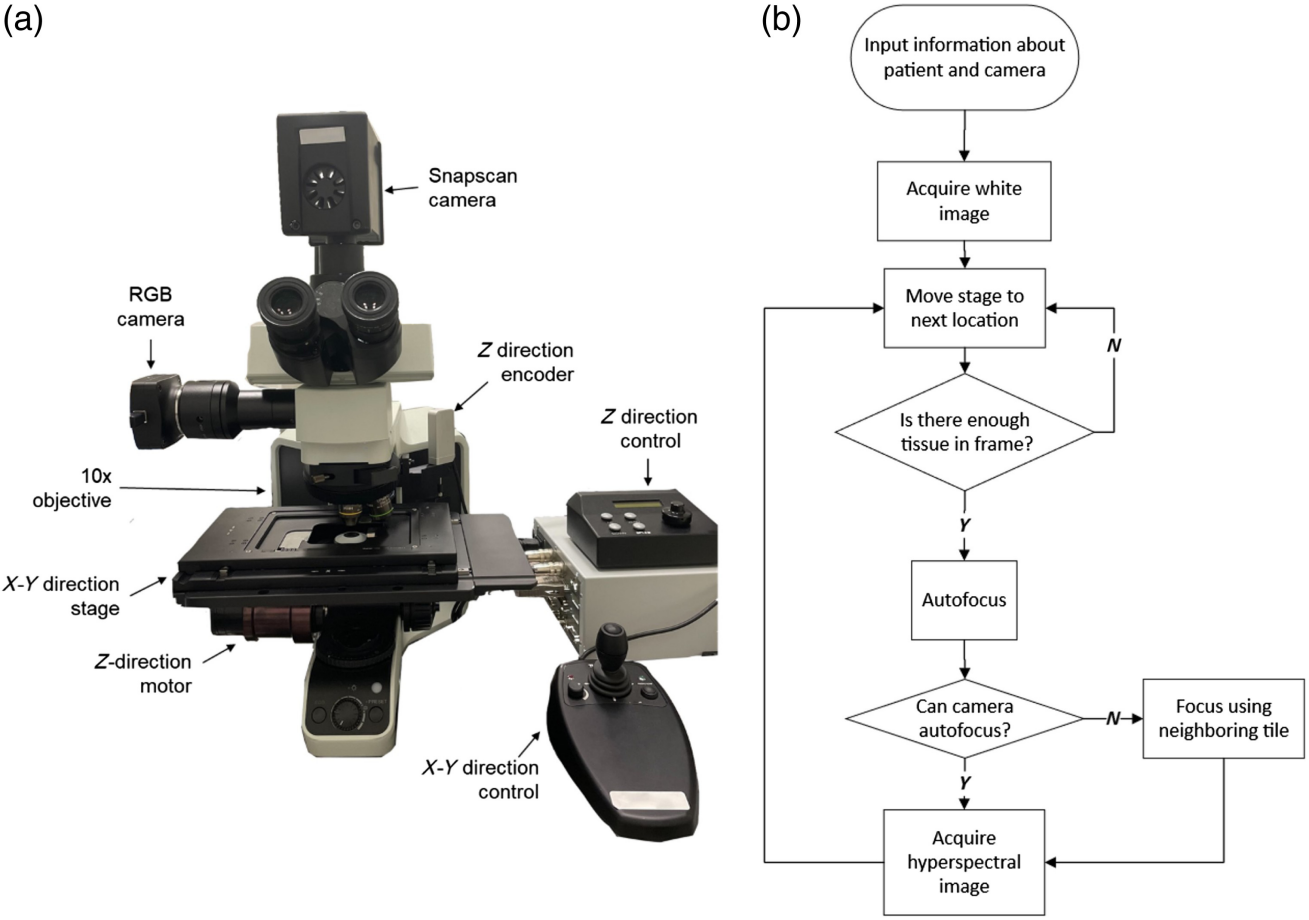
Overview of the acquisition system. (a) Photograph of the acquisition system with its components. (b) Flowchart of the automated acquisition software.

The system employed in this study is depicted in Fig. 1(a). It can automatically capture large WSHSIs. A more detailed description of the system is shown in our previous publication.^32^ The system consisted of an upright microscope (Olympus, Tokyo, Japan) and a customized hyperspectral snapscan camera that captures images by moving a sensor tiled with Fabry-Perot interferometers.^33^ The hyperspectral camera can image from 467 to 920 nm in 150 spectra, with a full width at half maximum value ranging from 3.1 nm (487 nm) to 11 nm (900 nm). The maximum image resolution was 2048×2048  pixels. The stage consisted of an X-Y motorized stage (Thorlabs, Newton, New Jersey, United States) and a Z-direction stepper motor (Prior, Rockland, Massachusetts, United States). The stage could be controlled in three spatial directions using the computer. A C++ program was developed to integrate all components together to scan hyperspectral images using the flowchart shown in Fig. 1(b).^32^ First, the stage and the camera identified the location of maximum focus using the Brenner filter.^34^ Then, the program moved the X-Y stage in a predetermined pattern and captured hyperspectral images.

### 2.2 Histological Specimen

The histological specimens consisted of 65 slides of thyroid cancer, which included three common types of thyroid carcinoma: papillary thyroid carcinoma (24 slides), follicular thyroid carcinoma (eight slides), and medullary thyroid carcinoma (three slides). The remaining 30 slides were normal non-carcinoma thyroid tissue. The specimen collection was described in our previous publication.^6^ The gross specimens were fixed, embedded, sectioned, and stained with H&E.

### 2.3 Hyperspectral Dataset

**Fig. 2:**
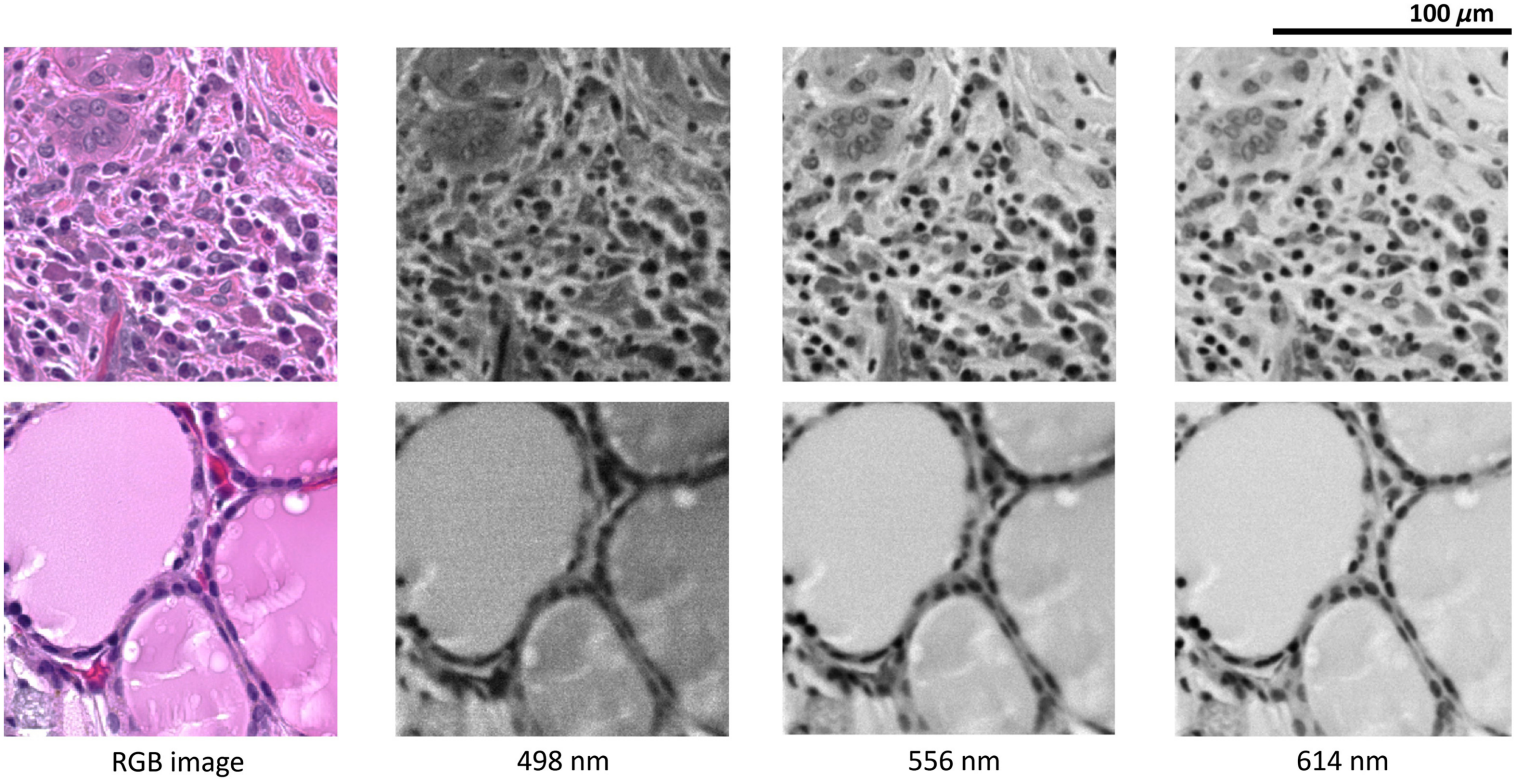
Example of hyperspectral images. Each image is 250×250  pixels and corresponds to a spatial field of size 139  μm×139  μm.

Using the instruments described in Sec. 2.1, we produced a dataset of 2599 images of size 2000×2000×84  pixels, with each image being 2000 pixels in length and width and each image containing 84 spectra from 467 to 721 nm. The size of the image was predetermined by the camera manufacturer’s settings. Each image was divided into non-overlapping sub-images of size 250×250×84  pixels (Fig. 2) and then resized into 224×224×84  pixels to fit into pretrained networks. The size of the sub-images was chosen to best balance between enough coverage of tissue structure and good margin accuracy. An anti-aliasing resizing technique was used to not introduce resizing artifacts. A 10× magnification objective was used, so each image had a spatial resolution of 0.556  μm/pixel.

Before the scanning of each slide, a white reference hyperspectral image was obtained at a blank area on the slide, and a dark current image was acquired automatically by the camera with the camera shutter closed. Afterward, each raw hyperspectral image was calibrated with the white reference and dark current images to obtain the transmittance of the tissue as

$$\text{Transmittance }(\lambda )=\frac{I_{\text{raw}}(\lambda )-I_{\text{dark}}(\lambda )}{I_{\text{white}}(\lambda )-I_{\text{dark}}(\lambda )},$$

where transmittance (λ) is the wavelength-dependent transmittance, $I_{\text{raw}}(\lambda )$ is the intensity value for wavelength λ in the raw hyperspectral images, and $I_{\text{white}}(\lambda )$ and $I_{\text{dark}}(\lambda )$ are the intensity values for wavelength λ in the white reference and dark current images, respectively. Each slide had an accompanying conventional whole-slide RGB image captured using a microscopic image scanner (Hamamatsu Photonics, Hamamatsu, Japan).^6^ From the whole-slide RGB images, a board-certified pathologist determined the gross margins and types of cancer digitally. These were the ground truths used for validation of the networks.

### 2.4 Transformer Architecture

**Fig. 3:**
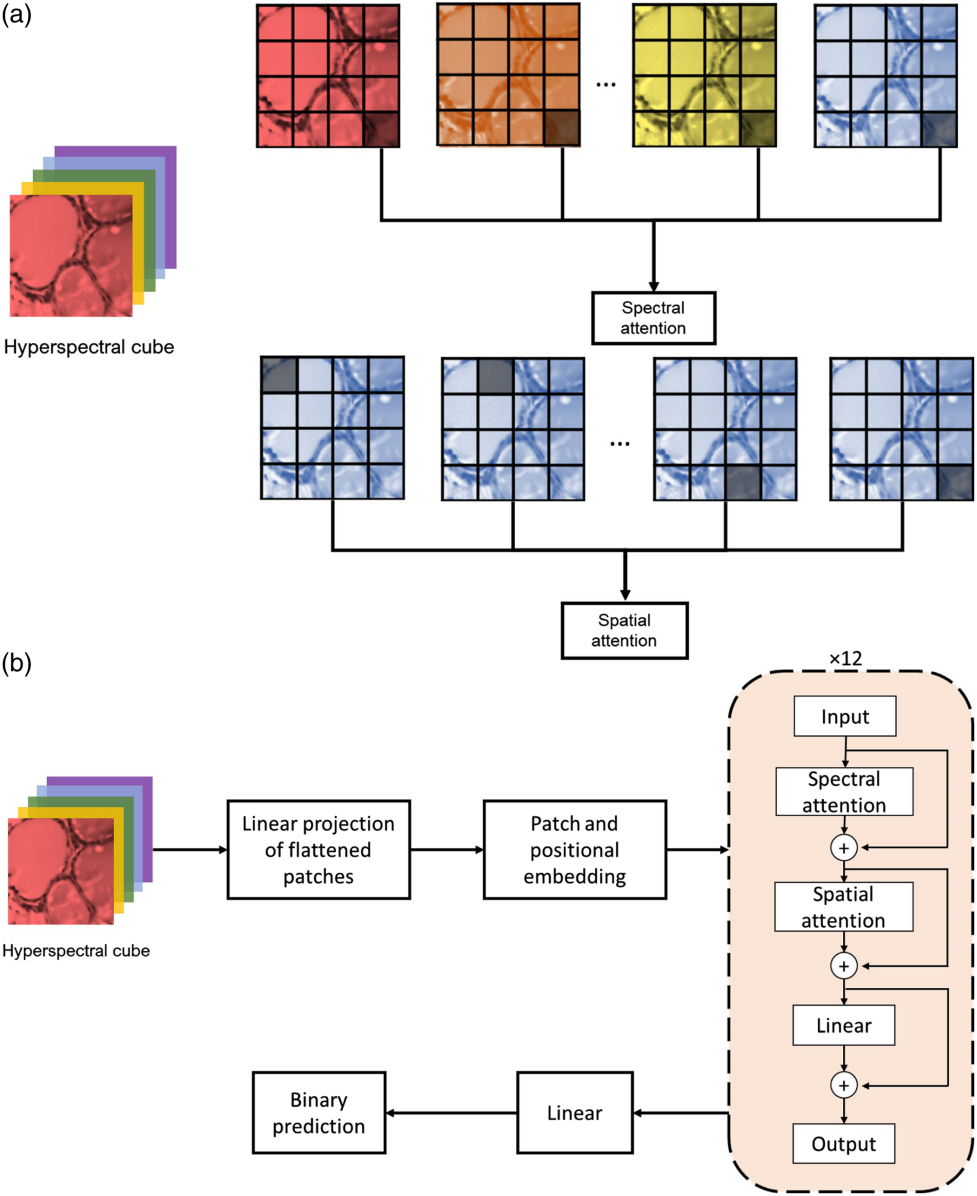
TimeSformer architecture modified for HSI. (a) Spatial attention compared the patch with other patches within the same wavelength band, whereas spectral attention compared the patch with other patches at the same spatial location but with different wavelengths. (b) The overall architecture for the TimeSformer network modified for HSI.

We used the “TimeSformer” transformer architecture that was described in our previous work.^35^ Transformers are multi-layer neural networks that use the mechanism of self-attention. In the self-attention mechanism, the input data (image, word sequence) is divided into small tokens. Each token has its own key-value pairs and its own query, so it can be matched with other tokens. The transformer network trains itself through gradient descent, so appropriate keys, values, and queries emerge. Queries can be multiplied with keys to produce a matrix of relevant weights, the result of which is retrieved as “values.” Within a transformer layer, they are represented using the attention equation, given as

$$\text{Attention }(Q,K,V)=\text{softmax}(QK^{T}/\sqrt{d})V,$$

where Q, K, and V are trainable weight matrices that correspond to the query, key, and value, respectively, and d stands for the length of the Q vectors. The SoftMax equation normalizes the elements $z_{i}$ within vector $z=[z_{1},z_{2},\ldots z_{i},\ldots ,z_{K}]$ using the equation $\text{softmax }(z)=\exp(z_{i})/\overset{K}{\underset{j=1}{\sum }}\;\exp(z_{j})$. The input images were first divided into 14×14 equal-size patches, and then, each patch was converted into a one-dimensional sequence using a convolutional kernel. Positional and class embeddings were used in a similar manner as with the ViT. The network consisted of 12 blocks, as shown in Fig. 3(b). Within each block, there were two separate attention layers: a spectral attention layer followed by a spatial attention layer. Figure 3(a) shows the graphical representation of spatial attention and spectral attention. In spectral attention, each patch was compared with other patches in similar spatial positions but at different wavelengths. In spatial attention, each patch was compared with other patches within the same spectral wavelength. This scheme of attention produced two different sets of keys, queries, and values matrices, one for spatial attention and one for spectral attention.

### 2.5 Augmentation of Spectral Data

**Fig. 4:**
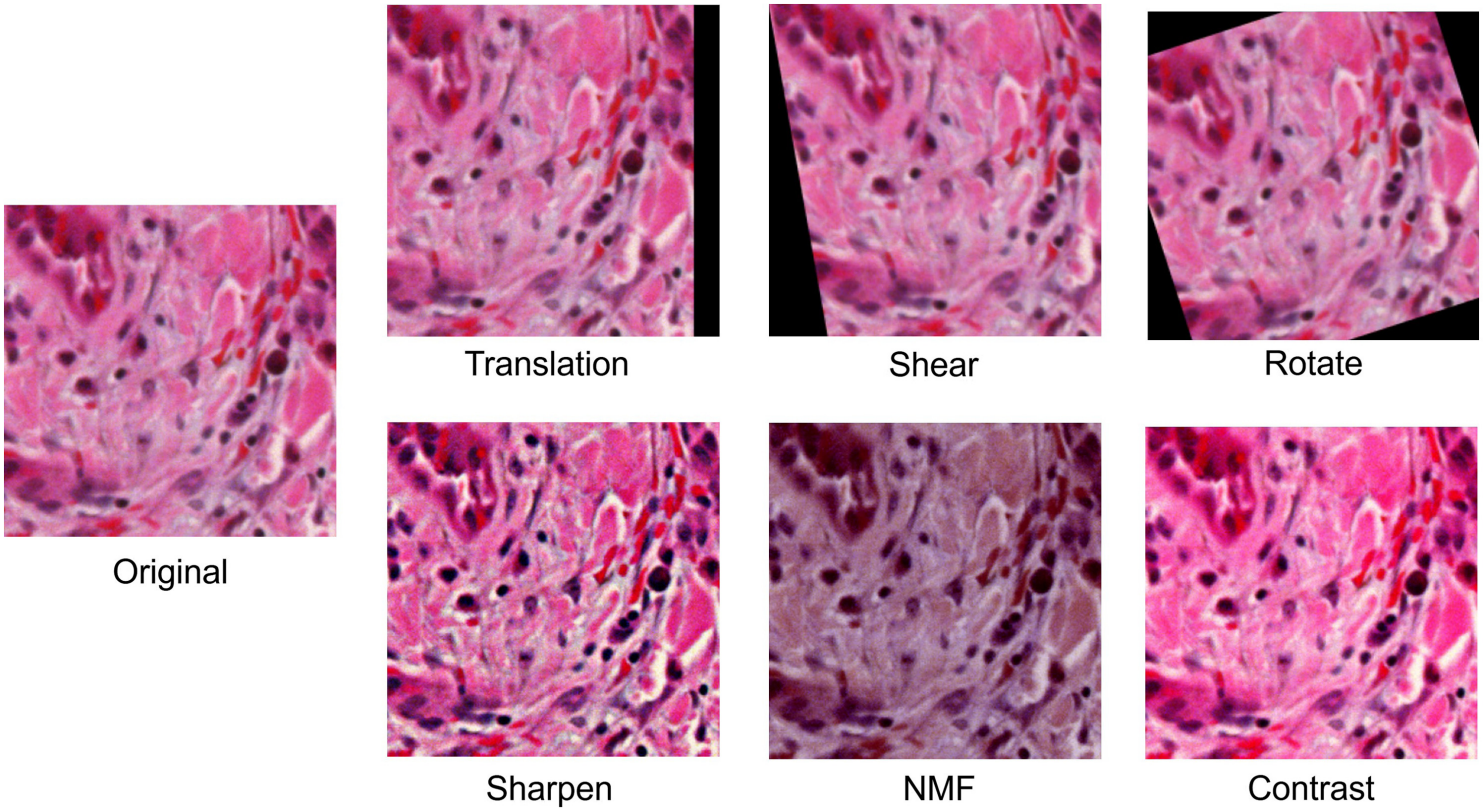
Effect of augmentations on the spatial domain of hyperspectral images. For visualization purposes, we applied transformation from HSI into RGB images using the method from Ma et al.^14^

**Fig. 5:**
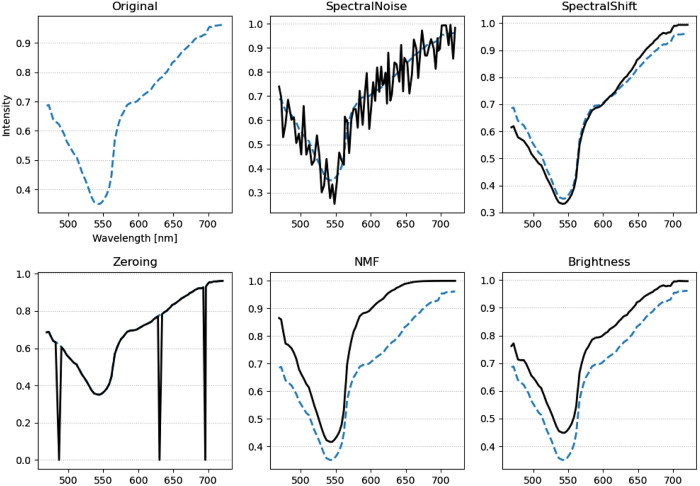
Effect of augmentations on the spectral domain of hyperspectral images. The spectra were generated by taking the spectrum average of the image patch. Blue dashed lines mark the original average spectra. Black solid lines mark the transformed average spectra.

Algorithm 1 shows the pseudo-code implementation of RandAugment for HSI. For each image, RandAugment successively applied three random transformations from the set of transformations.^29^ The strengths of the transformations were uniformly random values between the ranges shown in Table 1. In addition to the list of transformations seen in Faryna et al.^27^ (identity, rotation, shearing, translation, brightness, and contrast), we proposed the following tailored transformations: spectral noise, shifting, zeroing, and NMF estimation. We detail the specifics of each transformation. Rotation rotated the image by its center clockwise anywhere from 0 to π/2. Shearing sheared the image either horizontally or vertically from 0% to 50% of the image length. Translation translated the image either horizontally or vertically from 0% to 50% of the image length. Brightness increased the intensity of the image from 0% to 100% of the image’s maximum intensity. Contrast applied the contrast adjustment equation: $I^{\prime }=1.0157(C+1)(I-0.5)/(1.0157-C)+0.5$, where I, $I^{\prime }$, and C are the image before contrast enhancement, the image after contrast enhancement, and the contrast score, which ranges from 0 to 2.0, respectively.^36^ Sharpen applied the unsharp masking matrix, which can be scaled to increase or decrease the sharpness.^37^ Spectral noise applied a uniformly random shift to each image spectra that ranges from 0% to 50% of the maximum image intensity. Shifting introduced a linear deviation of the form $I^{\prime }(\lambda )=I(\lambda )A(\lambda -600\;\mathrm{nm})$, where I(λ) and $I^{\prime }(\lambda )$ are the spectra before and after modification that are dependent on wavelength λ, respectively, and A is the strength of the modification that ranges from 0 to 2. Zeroing randomly selected up to three channels and replaced them with black image bands. This augmentation method mimicked data occlusion schemes such as Cutout.^25^ NMF estimated the content of different tissue stains and modified them to simulate different stain concentrations.^36^ Using NMF, we decomposed the hypercube into three components, which corresponded to hematoxylin, eosin, and hemoglobin in the stained slide. The decomposed components were modified using gamma correction I′=I^γ, where I and $I^{\prime }$ are the image before and after modification, respectively, and γ is the gamma score that ranges from 0.5 to 1.5. We then reconstructed the hypercube from the decomposed cube. Table 1 shows the range of modifications applied for each transformation. We chose the values from a combination of experimental values and from values obtained by Faryna et al.^27^ Figure 4 shows the effect of augmentations in the spatial domain. Figure 5 shows the effect of augmentations in the spectral domain.

**Algorithm 1:** Hyperspectral RandAugment.

Transformations ← [Identity, Rotate, ShearX, ShearY, TranslateX, TranslateY, Brightness, Contrast, Sharpen, SpectralNoise, Shifting, Zeroing, NMF].
**for** i = 1 to 3 do
Randomly select a transformation
Image = Transform (Image, strength)

**Table 1:** Ranges of different types of data augmentation used. The intensity of an image is measured on a scale from 0 to 1, where 0 is the total black and 1 is the total white.

Transform	Range	Unit
Identity		
Rotate	[0,π/2]	Radians
Translate	[0, 0.5]	Image length
Shear	[0, 0.5]	Image length
Contrast	[0, 2.0]	Multiples of original contrast
Sharpen	[0, 5]	Sharpening multiplier
Spectral noise	[0, 0.5]	Intensity
Shifting	[0, 2.0]	Intensity
Zeroing	[0, 3]	Number of channels
Brightness	[0, 1.0]	Intensity
NMF	[0.5, 1.5]	Gamma correction unit

### 2.6 Training

To avoid data leakage, we used stratified sampling to split the data into training (28 slides), validation (seven slides), and test (26 slides) sets. Data was separated by patients, so no patients contributed to two splits. Table 2 shows the number of 250×250×84 images used for each dataset. In the training dataset, there were 47,630 images (56% of the training data) labeled normal and 37,627 images (44% of the training data) labeled cancer. In the validation dataset, there were 10,273 images (47% of the validation data) labeled normal and 11,379 images (53% of the validation data) labeled cancerous. In the test dataset, there were 26,539 images (56% of the test data) labeled normal and 20,638 images (44% of the test data) labeled cancer.

**Table 2:** Number of images used for the training, validation, and test datasets.

	Normal	Cancer	Total
Train	47,630	37,627	85,257
Validation	10,273	11,379	21,652
Test	26,359	20,638	46,997
Total	84,262	69,644	153,906

TimeSformer networks were trained for 15 epochs using a batch size of 16 and an initial learning rate of $1\times 10^{-3}$. We initialized the model with the pretrained weights from Bertasius et al.^30^ The optimizer used was stochastic gradient descent with a Nesterov momentum value of 0.9 and a learning rate decay of 0.5 every five epochs. The epoch that achieved the maximum validation $F_{1}$ score was selected for testing. The training was implemented in PyTorch using graphic processing units on high-performance computers. We trained two TimeSformer networks, one with and one without the augmentation methods described in Sec. 2.5.

We trained three additional networks for comparison with TimeSformer: a ConvNext trained on HSI, a ConvNext trained on RGB images, and a ViT trained on RGB images. We modified the “patchify” block of ConvNext^38^ to expand the input kernel into 84 input channels. This technique of increasing the number of input layers in pretrained CNN to fit HSI is shown to be effective in classifying H&E-stained hyperspectral data.^11^^,^^14^ To train ConvNext on HSI, a batch size of 16 and an initial learning rate of $1\times 10^{-3}$ were used. All training data were augmented using the method described in Sec. 2.5. The RGB images were synthesized from HSI using the method by Ma et al.^14^ To train ConvNext and ViT on RGB images, a batch size of 64 and an initial learning rate of $1\times 10^{-3}$ were used. In all networks, we used stochastic gradient descent with a Nesterov momentum value of 0.9 and a learning rate decay of 0.5 every five epochs. For augmentation, we used the default RGB RandAugment transformations in the PyTorch library.

### 2.7 Evaluation Metrics

We compared the performances of each network on the task of identifying thyroid cancer per image. The networks classified an image as either containing or not containing cancer cells based on labels provided by a clinically trained pathologist. For each model, the accuracy, the $F_{1}$ score, the AU-ROC, and the sensitivity score (also known as recall) were calculated. We then tested the performance of each network in identifying the cancer margin. The test dataset consisted of nine WSHSIs. To generate the cancer margin for each whole-slide image, we performed morphological opening (erosion followed by dilation) on each prediction. The metrics used to evaluate margins were the Jaccard index (intersection over union) and the Hausdorff distance. The Jaccard index J(A,B)=|A∩B|/|A∪B| calculates the intersection between two sets A and B over their union. The Hausdorff distance measures the largest distances between two morphological segmentations:

$$d_{H}(X,Y)=\max\{\underset{x\in X}{\sup}\;d(x,Y),\underset{y\in Y}{\sup}\;d(X,y)\},$$

where sup f refers to the supremum of function f and $d(a,B)=\underset{b\in B}{\inf}\;d(a,b)$ refers to the infimum of the distances from points a∈X to points b∈B.

### 2.8 Attention Unrolling

We visualized the weights of the trained attention modules using the attention unrolling technique.^19^ This method aggregates all attention weights across all layers into one. At each layer L, a single head produced an attention map $A_{h,L}$, which was the SoftMax multiplication of the keys $K_{h,L}$ and queries $Q_{h,L}$. The attention maps were averaged across all heads and added with the identity matrix I to produce the layer attention matrix A^L. The rollout attention was calculated as the dot product of all attention maps at all layers.

$${\text{Attention}}_{h,L}=\text{softmax}(Q_{h,L}K_{h,L}^{T})$$

A^L=I+average(attentionL),

Rollout=A^1⊙A^2⊙…⊙A^12.

The rollout attention matrices were scaled from 0 to 1 to produce attention heatmaps that showed the attention that TimeSformer paid to different biological features at different wavelengths.

## 3 Results

### 3.1 Classification Results

TimeSformer trained on the HSI dataset achieved an accuracy score of 90.87%, an $F_{1}$ score of 89.79%, a sensitivity score of 91.50%, and an AU-ROC score of 97.04%. Without data augmentation, TimeSformer achieved an accuracy score of 88.23%, an $F_{1}$ score of 86.46%, a sensitivity score of 85.53%, and an AU-ROC score of 94.94%. ConvNext trained on the HSI dataset achieved an accuracy score of 90.38%, an $F_{1}$ score of 88.74%, a sensitivity score of 86.27%, and an AU-ROC score of 96.23%. ViT trained on a synthesized RGB dataset achieved an accuracy score of 89.98%, an $F_{1}$ score of 88.14%, a sensitivity score of 84.77%, and an AU-ROC score of 96.17%. ConvNext trained on a synthesized RGB dataset achieved an accuracy score of 89.31%, an $F_{1}$ score of 87.84%, a sensitivity score of 87.91%, and an AU-ROC score of 95.63%. Table 3 shows that TimeSformer achieved the highest accuracy, $F_{1}$ score, sensitivity, and AU-ROC score of all networks on the test dataset. The results achieved by TimeSformer on the thyroid dataset are greater than those achieved by Halicek et al.^6^ on the same set of H&E-stained thyroid slides, which achieved an accuracy of 89.4% and an AU-ROC score of 95.4%.

**Table 3:** Accuracy, $F_{1}$ score, sensitivity, and AU-ROC scores for the test dataset.

Dataset	Network	Test metrics (%)			
		Acc	$F_{1}$	Sen	AU-ROC
HSI	TimeSformer (with augmentation)	**90.87**	**89.79**	**91.50**	**97.04**
	TimeSformer (no augmentation)	88.23	86.46	85.53	94.94
	ConvNext-HSI	90.38	88.74	86.27	96.23
S-RGB	ViT-RGB	89.98	88.14	84.77	96.17
	ConvNext-RGB	89.31	87.84	87.91	95.63

Data in bold means the highest score out of the four networks. HSI, hyperspectral image dataset; S-RGB, synthesized RGB image dataset; Acc, accuracy; Sen, sensitivity (recall).

### 3.2 Cancer Margin Detection in Whole-Slide Images

Out of the four networks tested, TimeSformer achieved the overall best average Hausdorff distance of 0.76  mm±0.33  mm, with a maximum of 1.47 mm and a minimum of 0.44 mm. In comparison, ConvNext trained on HSI achieved an average Hausdorff distance of 1.07±0.73  mm, ViT trained on RGB achieved an average of 0.99±0.64  mm, and ConvNext trained on RGB achieved an average of 1.08±0.52  mm. The results showed the ViT performs better for hyperspectral images. Table 4 shows the Hausdorff distance achieved on each slide. The table shows that typically slides that achieved good performances in TimeSformer also achieved good performances on other networks, and vice versa. The table also shows that TimeSformer produced good margins more consistently compared with other networks, as evidenced by the lower standard deviation value.

**Table 4:** Performance in terms of the Hausdorff distance on each slide. Data in bold refer to the network with the best performance.

Slide	TimeSformer	ConvNext-HSI	ViT-RGB	ConvNext-RGB
1	**0.65**	0.69	0.61	1.38
2	1.47	1.52	**1.46**	1.47
3	**0.69**	2.42	1.81	1.28
4	0.50	0.49	**0.34**	0.40
5	**0.44**	0.54	0.63	0.63
6	0.58	0.52	0.55	**0.51**
7	0.67	**0.61**	1.15	1.16
8	0.78	0.80	**0.35**	0.87
9	**1.10**	2.01	2.01	2.02
μ±σ	**0.76 ± 0.33**	1.07 ± 0.73	0.99 ± 0.64	1.08 ± 0.52

**Fig. 6:**
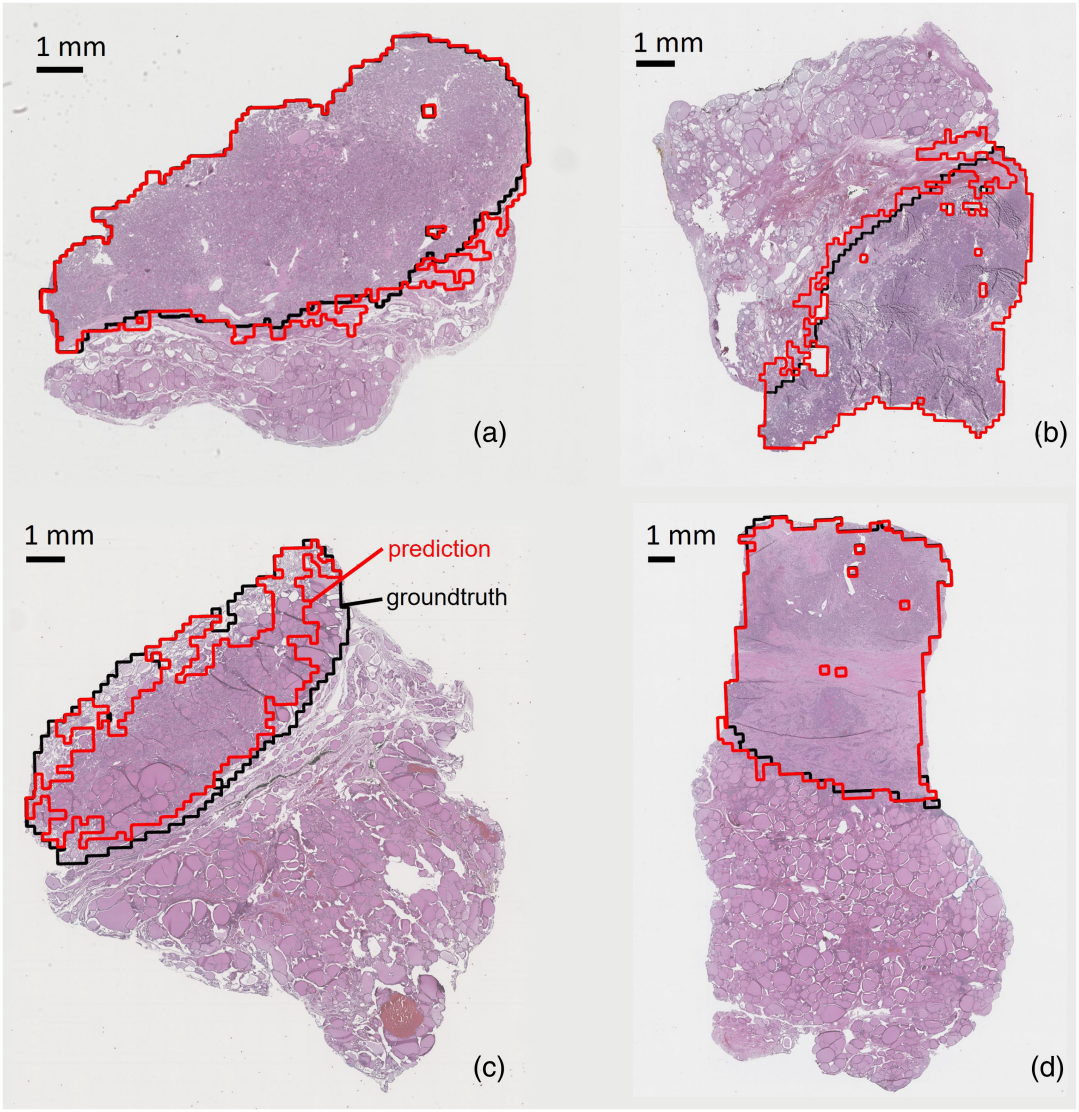
Cancer margins predicted on four thyroid whole slides. The black border shows the predictions made by the pathologist, and the red border shows the predictions made by TimeSformer. Whole-slide images were captured by a whole-slide histology scanner. (a) Whole-slide image of thyroid diagnosed with papillary carcinoma. (b) Whole-slide image of thyroid diagnosed with follicular carcinoma. (c) Whole-slide image of thyroid diagnosed with follicular carcinoma. (d) Whole-slide image of thyroid diagnosed with papillary carcinoma. The RGB images were taken using a whole-slide histology scanner.

Figure 6 shows the carcinoma margins in four representative slides. Figure 6(a) depicts slide 7, which contains papillary thyroid carcinoma. In this slide, the network achieved a Hausdorff distance of 0.67 mm and a Jaccard score (intersection over union) of 0.95. Figure 6(b) depicts slide 1, which contains follicular thyroid carcinoma. In this slide, the network achieved a Hausdorff distance of 0.65 mm and a Jaccard score of 0.88. Figure 6(c) depicts slide 2, which is a follicular carcinoma slide and the slide with the worst performance. Examination of the slide reveals that the classifier associated some fibrous structures with normal tissues, which might not be accurate for this particular instance. Figure 6(d) depicts slide 5, which is a papillary carcinoma slide and the slide with the best performance. Examination of the slide reveals that other networks also achieved good results on slide 5 and that slide 5 has a very clear delineation between carcinoma and normal tissues.

### 3.3 Visualization of Attention in Transformer

**Fig. 7:**
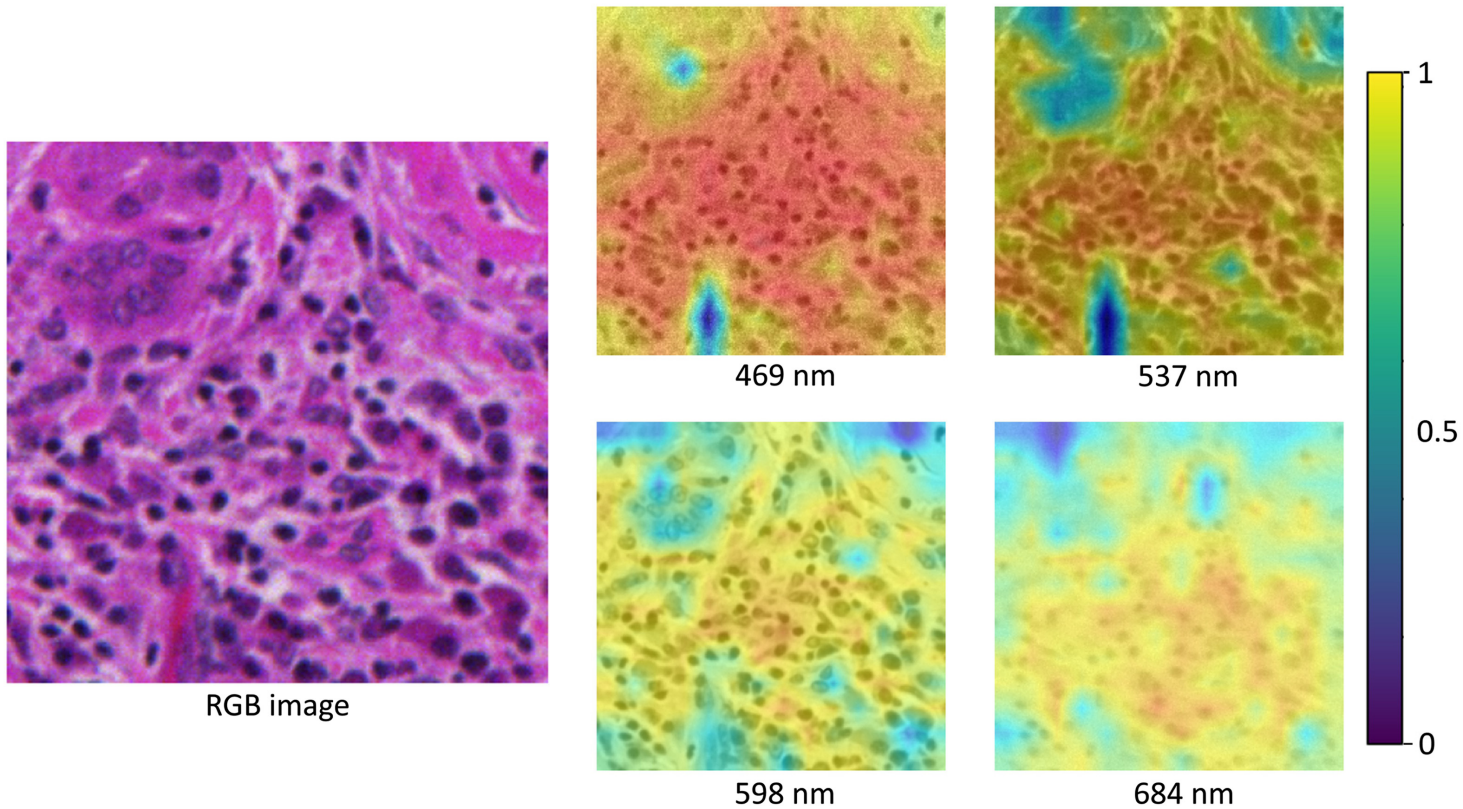
Attention map of an image that showcased papillary thyroid carcinoma. The RGB image was taken using a whole-slide histology scanner.

Figure 7 shows the spatial attention map of the trained network on an image of papillary thyroid carcinoma, for which the network produced an accurate prediction with a confidence of 96.51%. It could be seen that spatial attention was highest among the clusters of nuclei in the middle section; this attention was consistent across all wavelengths and highest in the blue-green wavelengths (470 to 540 nm). We attribute this to the fact that hematoxylin stains the nucleus blue, so nuclei were highlighted for the ViT. Our findings were consistent with the findings of Halicek et al.,^6^ which showed that deep neural networks focus on nuclei to predict the probability of cancer.

**Fig. 8:**
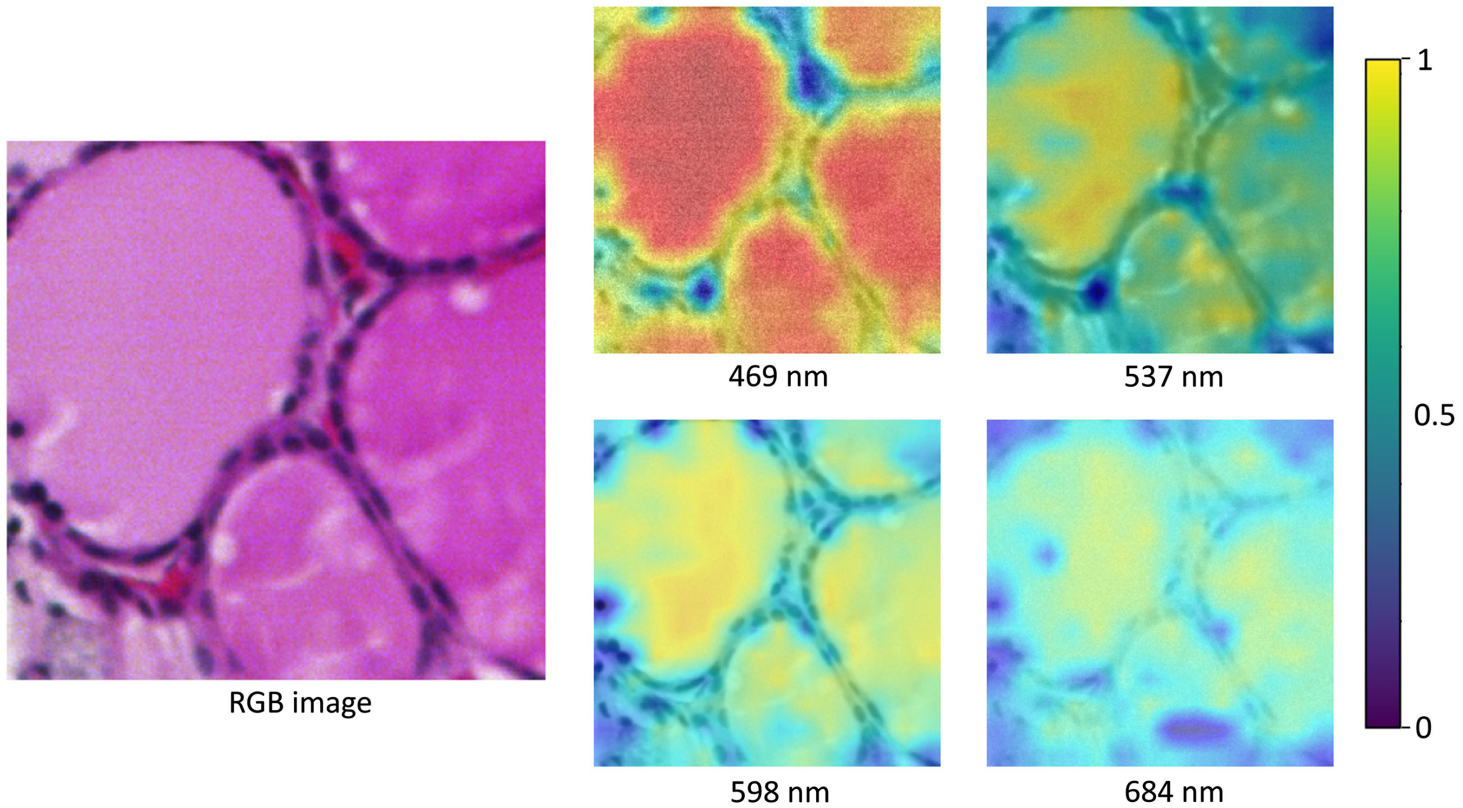
Attention map of an image that showcased regular healthy thyroid. The RGB image was taken using a whole-slide histology scanner.

Figure 8 shows the attention map of the trained network on a patch of healthy and normal thyroid cells. The network produced a correct prediction with 99.53% confidence. It could be seen that spatial attention was highest in the regions of follicular colloid, which are the fluid-filled sections of the thyroid follicle. Less attention was paid to the surrounding follicular thyroid cells. Spatial attention was consistent across all wavelengths and highest in the blue-green wavelength bands (470 to 540 nm).

## 4 Discussion and Conclusion

In this paper, we trained the TimeSformer network for the hyperspectral image dataset and developed hyperspectral augmentation methods to improve the classification performance. To that end, we showed the hyperspectral advantage in cancer margin detection on H&E slides of thyroid cancer patients. We trained HSI and RGB images on different machine learning networks. Using TimeSformer with augmentation, we achieved improvements in all metrics compared with the state-of-the-art networks on RGB data. We believe that such an improvement is generally difficult to achieve within the field of machine learning and that the metrics improvements bring clinical improvements. Deep learning methods can have tradeoffs between sensitivity and specificity, so a method that improves all metrics can be generally difficult.^6^ In terms of clinical applications, improved accuracy and sensitivity leads to better diagnosis with more confidence. Furthermore, we believe that TimeSformer is more consistent compared with existing machine learning networks, which translates to more consistent clinical diagnosis for patients.

The TimeSformer achieved the best mean outcome with an average Hausdorff distance of 0.76  mm±0.33  mm. This result was an improvement over that of Ma et al. on the same dataset^13^ and is comparable to that of existing literature.^39^^–^^41^ However, it is important to acknowledge that the mean Hausdorff distances for all algorithms overlap significantly within these uncertainties. This overlap indicates that, although TimeSformer has the best average performance, it may not consistently outperform the other algorithms in every instance. From a practical standpoint, the consistent performance of TimeSformer, as indicated by its lower standard deviation, is advantageous. In clinical applications, for which reliability and consistency are paramount, an algorithm that performs well across a wide range of scenarios is often preferred. The average Hausdorff distance was less than 1 mm, with the highest being less than 2 mm. This value is considered reasonable within clinical use.^4^^,^^13^ Slides that performed well with TimeSformer also tended to show good performance with other networks, indicating a correlation in performance across different algorithms for the same slides. This suggests that certain slides inherently pose more or fewer challenges regardless of the algorithm used. Although TimeSformer generally achieves the best average performance, there are instances in which other networks may outperform it on specific slides. This underscores the importance of evaluating multiple metrics and considering the context of each image when assessing algorithm performance. The real-world implications of adopting our algorithm are significant. More accurate and consistent margin assessments can lead to better surgical planning, ensuring that tumor margins are accurately identified and excised, which is critical for patient outcomes. Improved margin detection reduces the likelihood of residual tumor cells being left behind, thereby decreasing the chances of recurrence and the need for additional surgeries. We anticipate that its implementation could streamline the workflow in clinical settings, providing pathologists with a reliable tool to assist in margin assessment. This could lead to more efficient and accurate diagnoses, ultimately benefiting patient care.

The findings from the augmentation methods further substantiate the hyperspectral advantage. Spatial augmentations likely increased the invariance of the models to shifts and rotations, whereas spectral augmentations likely boosted the model’s ability to recognize relevant spectral features. These augmentation techniques may have contributed to the robustness of our models to variations and anomalies, improving their generalizability on the validation and test datasets. Although our results are promising, we acknowledge that further validation is required before widespread clinical adoption. Our current evaluation is based on a limited dataset, and it is essential to test the algorithm on a larger and more diverse set of hyperspectral whole-slide images. This will help ensure that the algorithm’s performance is robust and generalizable across different types of tissues and imaging conditions.

Our experiments showed that both TimeSformer and ConvNext trained on HSI outperformed ViT and ConvNext trained on RGB images. Our findings were in line with our previous work, which used a Visual Geometry Group (VGG)-19 network to train on HSI data and found improvements in AU-ROC compared with that of a VGG-19 network trained on RGB images.^11^^,^^14^ It was also similar to the findings by Sun et al.^17^ that found improvements on the same networks when trained on a hyperspectral image dataset compared with on RGB images. This could be unintuitive at first as H&E stains have few well-known peaks, so additional wavelengths may not provide additional information. However, we believe that hyperspectral images provide an advantage over RGB images because it can increase the contrast between stains and dyes. More research needs to be done to identify potentially useful wavelengths related to H&E stain classification.

Our study involves visualizing and unrolling the attention layers of the TimeSformer model. The process of unrolling refers to breaking down the attention patterns at each temporal dimension. In this way, we hoped to discern how attention weights evolve across the spectrum. To our knowledge, this represents the first work that uses ViT visualization to reveal biological features of interest in thyroid histopathology. Although the TimeSformer architecture allows for a robust temporal understanding of hyperspectral images, the interpretability of its attention mechanism poses significant challenges. Visualizing attention scores can be misleading, primarily because the correlation between high attention weight and feature importance is not always straightforward.^42^ One might incorrectly infer that positions with lower attention weights are less critical, whereas these positions might be influential when interacting with other positions or through multiple layers of attention. Although previous literature has highlighted nuclei as key points of attention, our study uniquely identifies follicular colloids as regions of significant attention. This novel finding requires careful consideration as it may suggest new avenues for understanding the pathology of thyroid conditions. The broader implications of this attention are multifaceted. Consistent identification of nuclei and follicular colloids could imply that the algorithm is robust, leveraging these features as reliable indicators for distinguishing cancerous from non-cancerous regions. This robustness is particularly advantageous in clinical applications, for which consistent and reliable features are essential for accurate diagnosis. However, the focus on these features also raises potential challenges. If nuclei and follicular colloids appear inconsistently across different images, this could introduce variability in the algorithm’s performance, potentially hindering its reliability in a broader range of clinical scenarios. Therefore, further research is necessary to investigate the consistency of these features across diverse datasets and their overall impact on the algorithm’s effectiveness. It is essential to consider attention unrolling to be just one of many tools to interpret these complex models. Future work could involve developing more reliable methods of interpreting attention or exploring other aspects of the TimeSformer model.

## Data Availability

Code and data underlying the results presented in this paper are not publicly available at this time but may be obtained from the authors upon reasonable request.
